# On growth and flow: hydraulic aspects of aboveground meristems

**DOI:** 10.1111/nph.70713

**Published:** 2025-11-16

**Authors:** Juan Alonso‐Serra

**Affiliations:** ^1^ Organismal and Evolutionary Biology Research Programme, Faculty of Biological and Environmental Sciences and Viikki Plant Science Centre University of Helsinki Viikinkaari 1 Helsinki 00790 Finland

**Keywords:** biomechanics, cambium, hydraulics, meristem, shoot apical meristem, water

## Abstract

Water is essential for plant growth under both normal and stress conditions. Aboveground, two key meristems control plant development: the shoot apical meristem and the vascular cambium. Here, stem cell maintenance and cell differentiation are affected by hydraulic fluctuations across seasons, days, or even hours. Water fluxes, turgor pressure, osmotic gradients, and tissue mechanics are integrated by molecular signals to provide a robust control of meristematic activity. Despite this fundamental connection, our understanding of how meristems sense and respond to hydraulic changes is only beginning to emerge. Thus, integrating insights from research on plant stress and development opens exciting avenues to study meristem plasticity.

**Table:** 

	Content	
	Summary	722
I.	Introduction	722
II.	Hydraulic and mechanical properties of the SAM	723
III.	Developmental and molecular responses to water deficit at the SAM	724
IV.	Hydraulic and mechanical properties of the cambium	724
V.	Developmental and molecular responses to water deficit at the cambium	726
VI.	Conclusions and outlook	726
	Acknowledgements	726
	References	726

## I. Introduction

Meristems play a fundamental role in plant development, serving as a stem cell reservoir, a source of differentiating tissues, and hubs for developmental plasticity. Aboveground, two meristems play a major role in these processes: the shoot apical meristem (SAM) and the vascular cambium. The SAM is responsible for new organ formation and longitudinal growth, while the cambium produces vascular tissues, driving the plant's secondary growth.

In these meristems, growth rate and timing (e.g. seasonality) are constrained by internal and environmental water conditions. In essence, cell growth depends on its capacity to attract water (its water potential), which is linked to its turgor pressure (the pressure of water against cell walls), its osmotic pressure (osmolyte concentration), and its accessibility to water (e.g. tissue conductivity). These variables are ultimately governed by the plant water status, which depends on environmental water availability. Indeed, multispecies studies show that water availability is the most significant environmental factor determining cambial growth (Cabon *et al*., 2022). The SAM is also sensitive to water conditions, but due to its multiple developmental outputs – including organ formation, reproduction, branching, and partly, stem elongation – the impacts of water stress on this meristem are often difficult to dissect (Gray & Brady, 2016). On a shorter time scale, the growth rate in aboveground plant organs correlates with diurnal water status fluctuations, and cambial growth appears to follow this pattern too (Zweifel *et al*., 2021). Therefore, meristematic activity is influenced by stress and non‐stress, and short‐ and long‐term hydraulic fluctuations. Although our understanding of how meristems respond to these changes at the molecular and physiological level is limited, research on plant stress responses provides valuable insights into how altered water conditions may impact meristem function.

## II. Hydraulic and mechanical properties of the SAM

**Fig. 1:**
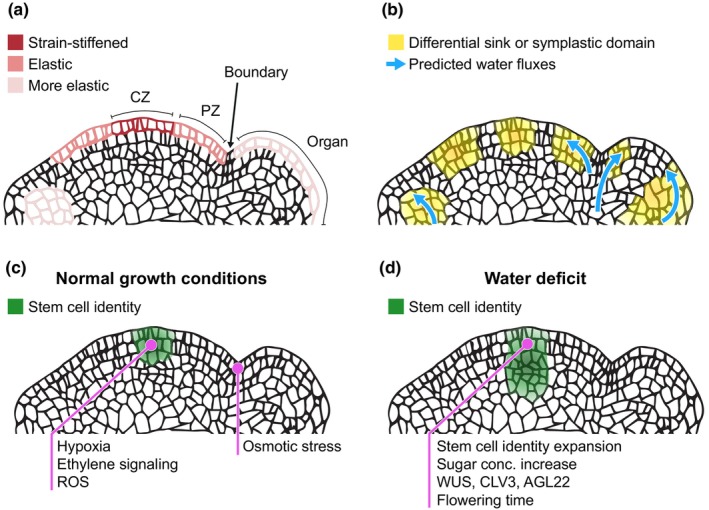
Hydraulic and molecular characteristics of the shoot apical meristem (SAM). (a) Mechanical properties of meristematic cell walls across different SAM domains: central zone (CZ), peripheral zone (PZ), boundary, and the organ. (b) Dye tracing experiments highlight distinct sink or symplastic domains in the SAM. In incipient organs, dyes allocate to deeper cell layers where cell walls are more elastic (see a). Water fluxes (blue arrows) are directed to fast‐growing cells in the organ and PZ, and away from the boundary domain. (c) Stem cell identity cells (defined by CLV3 expression) are localized in the top 3 cell layers of the CZ. Under normal growth conditions, the stem cell population is maintained by stress‐like signals (hypoxia, ethylene, and reactive oxygen species (ROS)), while the boundary shows osmotic stress‐like responses. (d) Under water deficit, the stem cell identity domain expands. Water stress induces physiological (increased sugars), molecular (WUS, CLV3, and AGL22 expression), and developmental (altered flowering time) responses.

Tissue hydraulics and mechanics are tightly connected (Cheddadi *et al*., 2019), and as a consequence, epidermal SAM domains are differentially affected by osmotic treatments. The central zone (CZ), where stem cells are located, is more prone to shrinkage under hyperosmotic conditions. On the other hand, the peripheral zone (PZ), where organs emerge, is more elastic and swells under hypoosmotic conditions (Fig. 1a) (Kierzkowski *et al*., 2012). These experiments highlight the varying elastic properties of cell walls and suggest a differential sensitivity to deformation across SAM domains. Cell wall differences, such as level of pectin methyl‐esterification (Peaucelle *et al*., 2008) or pattern of β‐1,4‐galactan (Yang *et al*., 2016), may partly contribute to such variation. At the organ primordium, cell walls are more elastic compared to the meristematic dome, and turgor pressure is predicted to be lower, assuming that the osmotic pressure remains similar (Peaucelle *et al*., 2011; Cheddadi *et al*., 2019).

At the CZ‐PZ epidermal cell layer, turgor pressure is heterogeneous, which appears to arise from variations in cell size and neighbor number rather than osmotic differences: smaller cells with fewer neighbors have higher turgor and growth rates than larger ones (Long *et al*., 2020). While osmotic pressure has not been directly measured, plasmolysis experiments suggest no detectable differences in this domain (Long *et al*., 2020). Such a hydraulic context would be expected to generate water potential heterogeneities and thus water fluxes. However, water movement driven mainly by turgor differences is inherently coupled to cell volume changes, making these fluxes ultimately rate‐limited by cell growth. It remains possible that minor osmotic pressure and hydraulic conductivity differences contribute to equilibrating the water potential heterogeneity. In the future, direct measurements of osmotic pressure and water fluxes, including those that might occur without cell volume change, will be crucial to resolve how such dynamic hydraulic heterogeneity is maintained at the SAM.

The fact that organs grow implies that they can attract water and have a lower water potential compared to other SAM domains (Cheddadi *et al*., 2019). Indeed, the higher volumetric growth in emerging organs acts as a differential water sink, potentially patterning how water moves in the SAM (Alonso‐Serra *et al*., 2024). Unlike carbon sinks, which are linked to both growth and storage, water sinks in incipient organs are transient and tied to cell expansion. They decline once growth stops and resume later with transpiration demand. One consequence of this differential sink strength at early developing organs is the shrinkage of neighboring boundary cells (Fig. 1b). These volumetric fluctuations involve water movement but should be interpreted cautiously, as they only reflect the net balance of water moving in and out of the cell. So far, water movement in small tissues like the SAM has not been directly assessed. Instead, water‐soluble dyes have been used to indirectly assess the spatiotemporal pattern of symplastic fields. Microinjection experiments with Lucifer Yellow have shown that in birch trees, the CZ and PZ conform to partially distinct symplastic domains (Rinne & Van der Schoot, 1998). This symplastic conductivity is reduced by growth cessation stimuli such as short photoperiod and restored by growth reactivation conditions such as cold temperature (Rinne *et al*., 2001). In Arabidopsis, the movement of water‐soluble HPTS (8‐hydroxypyrene‐1,3,6‐trisulfonic acid trisodium salt) from leaves to the SAM is also conditioned by photoperiod (Gisel *et al*., 1999, 2002). After reaching the SAM, HPTS localizes to the outer three cell layers, while deeper cells are less accessible, especially in the CZ. Organs show a higher dye signal, even before the vasculature is established, which is consistent with the differential sink strength model (Fig. 1b) (Gisel *et al*., 1999; Alonso‐Serra *et al*., 2024).

Dye allocation patterns result from two variables difficult to decouple: sink strength and tissue accessibility. While growth‐driven water movement is a passive physiological process, tissue accessibility can be additionally controlled by molecular signals. Plasmodesmata (PD) connections pattern symplastic fluxes at the SAM and PD aperture is modulated by abscisic acid (ABA) (Tylewicz *et al*., 2018; Wu *et al*., 2023). Soil water conditions modulate PD aperture in the root (Mehra *et al*., 2022), but symplastic connectivity in the SAM under water deficit conditions remains unexplored. Low air humidity induces ABA biosynthesis and long‐distance transport (Rowe *et al*., 2023), potentially reaching the SAM too. In ABA‐deficient plants, the HPTS pattern at the SAM is significantly disrupted, leaking into the apoplast (Alonso‐Serra *et al*., 2024). While PD closure is likely compromised in this mutant, such a pattern may emerge from the pleiotropic effects of ABA deficiency, such as membrane leakage (Waszczak *et al*., 2024). Furthermore, since ABA modulates plants' water status systemically, it can increase or decrease the hydraulic conductivity of plant tissues depending on the experimental conditions (Tardieu *et al*., 2010). In axillary meristems, PD aperture in adjacent phloem tissue is required for overall meristematic growth (Paterlini *et al*., 2021). A few PD‐related proteins also showed a degree of domain‐specificity at the SAM (Bayer *et al*., 2008), but the extent to which local symplastic domains are required for normal development remains unresolved. Aquaporins might likewise pattern symplastic‐apoplasmic fluxes in the SAM, yet their role has not been investigated. Taken together, these studies highlight several research opportunities in SAM hydraulics, but meristem‐specific manipulations would be ultimately required to decouple local and systemic effects.

## III. Developmental and molecular responses to water deficit at the SAM

Water stress is one of the most extensively studied abiotic stress factors. In recent years, the developmental plasticity of roots has gained significant attention, driven by substantial progress in understanding the underlying hydraulic dynamics, as well as transcriptional, post‐transcriptional, hormonal, and mechanosensing signaling mechanisms (Robbins & Dinneny, 2018; Shkolnik *et al*., 2018; von Wangenheim *et al*., 2020; Mehra *et al*., 2022).

At the SAM, water stress responses converge on two key aspects: carbon allocation and meristematic maintenance (Fig. 1c,d). Growth in younger organs (e.g. leaves) is more constrained by a carbon limitation than by a hydraulic demand (Pantin *et al*., 2011), and it is tempting to speculate that the same trend extends to the SAM. In sunflowers, water deficit stimulates sucrose accumulation at the capitulum and leaves (Dosio *et al*., 2011), and similar responses were observed in Arabidopsis flowers and leaves (Valifard *et al*., 2024). Under normal water conditions, sugar promotes meristem maintenance at the shoot apex by promoting SHOOT MERISTEMLESS (STM) expression (Lopes *et al*., 2024). Similarly, induction of sugar glycolysis genes appears to be critical for SAM adaptation to elevated temperatures (Olas *et al*., 2021), indicating that increased sugar concentrations may constitute a physiological stress response shared by meristems and young organs. Further meristem‐specific studies on sugar dynamics in the SAM are required, as sugars may not only support growth reactivation but also act as osmolytes, lowering the water potential and preventing water loss in meristematic tissue.

Osmotic stress increases the expression level and enlarges the expression domain of key SAM regulators such as WUSHEL and CLAVATA3 (Zeng *et al*., 2021). In this line, plants overexpressing meristem identity genes are more tolerant to drought (Lee *et al*., 2016). After flowering, water deficit negatively impacts new organ production at the shoot apex, partly via ABA‐dependent regulation of STM (Su *et al*., 2013; Wang *et al*., 2024). Before flowering, drought affects developmental transitions at the SAM, notably by accelerating flowering as a stress‐escape strategy when plants are grown in long‐day conditions (Riboni *et al*., 2016). These findings highlight that the impacts of water deficit on the SAM depend on its developmental stage. In annual plants such as Arabidopsis, this is tightly linked to its reproductive strategy. Changes in turgor pressure may also affect the pattern of hormone fluxes in the SAM by impacting the amount and polarity of auxin transporters (Nakayama *et al*., 2012). Calcium signals may provide a link between SAM turgor fluctuations and PIN1 polarity (Li *et al*., 2019). Interestingly, growing evidence suggests that signals classically associated with stress responses play a crucial role in meristematic maintenance under normal growth conditions (Fig. 1c). Examples of these are reactive oxygen species (Zeng *et al*., 2017), hypoxia (Weits *et al*., 2019), ethylene signaling (Zeng *et al*., 2021), and osmotic stress (Alonso‐Serra *et al*., 2024). Furthermore, ABA and osmotic stress signals promote totipotency at the SAM (Wilson *et al*., 2016; Chen *et al*., 2021). Taken together, these findings suggest that the SAM also relies on stress‐like signals to function under normal conditions, where hydraulic and mechanical fluctuations shape meristematic growth.

## IV. Hydraulic and mechanical properties of the cambium

**Fig. 2:**
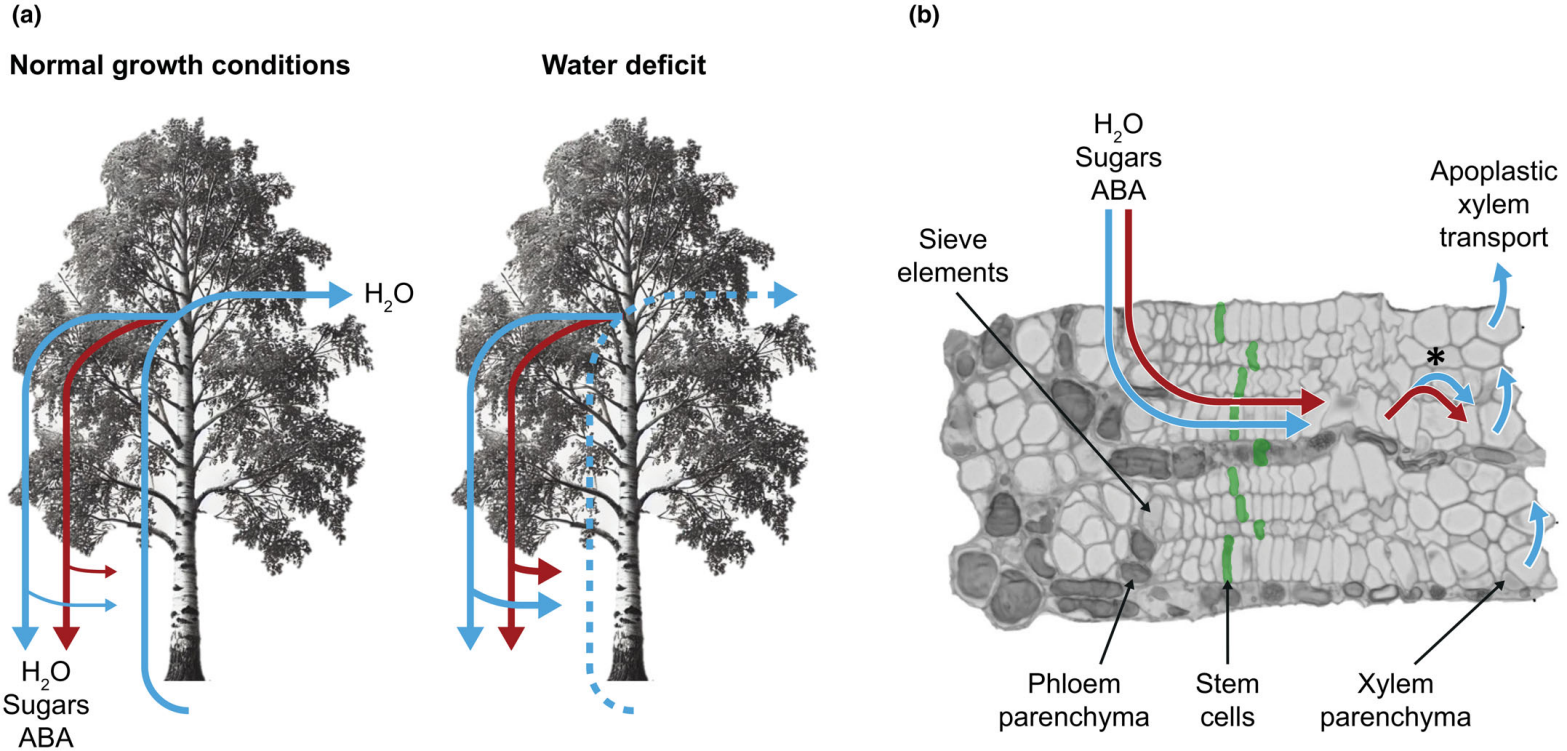
Hydraulic fluctuations at the cambium. (a) Under normal conditions, water (blue arrows) moves upwards through the xylem driven by evapotranspiration. It is then transferred to the phloem at carbon source tissues and is transported toward the roots along with sugars and hormones (red arrows). Under water deficit, xylem water transport is reduced (dashed line) and phloem unloading increases (as indicated by thicker arrows). ABA, abscisic acid. (b) Cambium anatomy showing the localization of cell types forming a symplastic continuum including phloem parenchyma, stem cells, and xylem parenchyma. Apoplastic transport in the xylem occurs through vessels. Under severe water deficit, water and sugars may move from xylem parenchyma into vessels, as indicated in the image with an asterisk (*).

A primary role of the cambium is the production of vascular tissues: xylem and phloem. Since xylem developmental plasticity is critical for coping with water stress, this tissue has been systematically studied in Arabidopsis (Thonglim *et al*., 2023) and trees (Rodriguez‐Zaccaro & Groover, 2019). Conversely, the hydraulic properties of cambial cells are less understood. During active growth, cambial cells undergo periclinal divisions resulting from the combined effects of tissue compression (Brown & Sax, 1962), circumferential tensile stress (Höfler *et al*., 2024), and centripetal diffusion of growth‐promoting signals (Etchells & Turner, 2010). Current hydraulic models suggest that water and sugar status contribute to determining the cambial turgor pressure required to enable growth (Hölttä *et al*., 2010; Peters *et al*., 2021; Potkay *et al*., 2022). However, cambial turgor pressure has not been directly assessed; instead, it is predicted using models or inferred from other hydraulic parameters. Small, yet detectable, stem diameter changes correlate with the stem turgor pressure and result from two processes: growth (cell division and mainly cell expansion), and reversible tissue deformation linked to water fluxes. Such stem diameter changes are observed under droughts, seasonal, and even diurnal water status fluctuations (Zweifel *et al*., 2021). Stem diameter, water potential, and turgor pressure are reduced during the day when the evaporative demand is at its highest, and restored during the night (Brunetti *et al*., 2019; Zweifel *et al*., 2021). Secondary growth, and more specifically xylem formation, is expected to coincide with periods of diameter increase (Zweifel *et al*., 2016), although direct evidence of hourly fluctuations in xylogenesis is missing. Droughts additionally result in increased sugar levels in bark and wood tissues, which may prevent further water loss and facilitate recovery upon rehydration (Brunetti *et al*., 2019, 2020). Potassium has also been proposed as an osmoregulator of cambial activity (Wind *et al*., 2004). Thus, secondary growth is directly influenced by changes in water fluxes and osmolyte concentration (Fig. 2a).

Water flow is *per se* a passive process and the translocation from the xylem to phloem tissues occurs primarily at carbon source tissues (e.g. leaves). Here, photoassimilates are loaded into phloem cells, reducing their water potential and drawing water in from the xylem. Water influx increases the phloem turgor pressure, driving a mass flow of water and sugars toward sink tissues such as the cambium. Cambial cells are symplastically connected to phloem and xylem parenchyma, which enables radial and axial transport of water, molecular signals, and sugars (Fig. 2b) (van der Schoot & van Bel, 1990; Spicer, 2014). Such symplastic and apoplastic connections have been proposed from dye tracing experiments and membrane potential measurements (van der Schoot & van Bel, 1990; Sokołowska & Zagórska‐Marek, 2012). Under drought conditions, water and osmolytes can be further transported from xylem parenchyma into vessels to facilitate embolism refilling (Secchi & Zwieniecki, 2016; Secchi *et al*., 2017). Interestingly, phloem osmotic pressure and bark thickness also show diurnal fluctuations under normal water conditions in conifers (Mencuccini *et al*., 2013). The above‐mentioned studies show that phloem and bark cells are important water and sugar sources during normal and stress conditions (Fig. 2b). These physiological responses are species‐dependent and indicate that stem water status alters carbohydrate movement, consequently modifying the hydraulic properties of vascular tissues.

The high symplastic conductivity between phloem parenchyma, cambium, and xylem parenchyma results in a three‐dimensional network linked via PD. In the cambium, PD numbers are transiently increased upon cell division, but they decrease as xylem cells mature. In addition, the PD numbers also vary during seasons, increasing in the spring when growth is activated and decreasing in the autumn (Fuchs *et al*., 2010). Thus, symplastic domains also show plasticity indicating that active control of tissue hydraulics may be present at the cambium, yet more studies are required to characterize how these might be regulated.

## V. Developmental and molecular responses to water deficit at the cambium

Prolonged water deficit leads to reduced growth rates and ultimately less biomass. However, since xylem developmental plasticity also relies on producing new stress‐adapted cells, cambial activity doesn't seem to stop immediately. Furthermore, cambial cell numbers show little to no changes under drought conditions while xylem vessel number increases, indicating changes in cell type specification (Arend & Fromm, 2007). ABA concentration in bark and xylem tissues increases under water stress, and it increases further during rewatering (Brunetti *et al*., 2019, 2020). Interestingly, ABA concentration in the bark can also fluctuate hourly without local changes in the expression of ABA biosynthesis genes (Brunetti *et al*., 2019). Such ABA dynamics under normal growth conditions mirror diurnal changes in a tree's water status, which appear to result from leaf‐sourced ABA, while stress conditions may induce additional ABA biosynthesis and transport in stem and root tissues. These hormonal dynamics suggest that sensing of water status at the cambium may be partly non‐cell‐autonomous and relies on leaf‐to‐stem ABA transport. It is tempting to hypothesize that this process may be linked to water fluxes, analogous to mechanisms previously described in roots (Mehra *et al*., 2022). When provided artificially, ABA can increase or decrease cambial cell numbers in trees depending on the tissue source (Fromm, 1997; Arend & Fromm, 2013). During the active growth season, ABA levels positively correlate with cambial cell numbers (Arend & Fromm, 2013), highlighting how stress signals are inherent to meristem maintenance. On the other hand, long‐term water stress reduces growth‐promoting signals such as cytokinin (Paul *et al*., 2018). Since xylem cells are specified within the cambial domain, the above‐mentioned hormonal fluctuations may also influence the rate, pattern, and type of xylem production, a well‐documented anatomical response to water stress. Together with cell differentiation changes outside the cambial domain, these molecular dynamics could ultimately shape xylem developmental plasticity. In future studies, sampling cambial tissue at higher spatial and temporal resolution would greatly enhance our understanding of the dynamic crosstalk between stress and growth signals.

## VI. Conclusions and outlook

Studies of plant developmental plasticity have significantly advanced our understanding of the molecular signals involved in stress acclimation. However, most research to date has focused on cell differentiation processes, while the strategies employed by stem cells and meristematic tissues remain less clear. Partly, this is due to the historical separation between the fields of plant stress and development, but also due to technical challenges in accessing aboveground meristems.

Despite these limitations, the integration of different sampling strategies has produced valuable insights into key mechanisms governing meristematic plasticity. First, in both meristems, there is a close interplay between osmolyte concentration, water fluxes, and growth. Sugars play a crucial role in this context both as a carbon source required for growth and as an osmolyte. Sugar transport repatterns the water potential of tissues, which can contribute to the direction and strength of water fluxes. Future research into meristem hydraulics would significantly benefit from identifying direct mechanisms linking physiological status, perception, and stem cell regulation. Turgor‐ or flux‐dependent signaling mechanisms are particularly interesting in this direction.

A second aspect emerging from the literature is that molecular signals previously linked to stress are inherently active in both meristems. This molecular signature might reflect the ubiquity of physical fluctuations during normal development, and as discussed previously, it contributes to meristem maintenance. Ultimately, while categorizing conditions as ‘stress’ or ‘non‐stress’ can be experimentally useful, plants likely perceive fluctuations across a continuous dynamic range, with a few critical thresholds that *we* define as stress. Thus, although plant stress responses and meristem biology are often studied independently, understanding plant stem cell behavior necessarily blurs the boundaries between these research fields.

## Competing interests

None declared.

## Disclaimer

The New Phytologist Foundation remains neutral with regard to jurisdictional claims in maps and in any institutional affiliations.
